# Lung neuroendocrine tumors: correlation of ubiquitinylation and sumoylation with nucleo-cytosolic partitioning of PTEN

**DOI:** 10.1186/s12885-015-1084-5

**Published:** 2015-02-20

**Authors:** Stéphane Collaud, Verena Tischler, Andrej Atanassoff, Thomas Wiedl, Paul Komminoth, Christian Oehlschlegel, Walter Weder, Alex Soltermann

**Affiliations:** 1 Division of Thoracic Surgery, University Hospital, Zurich, Switzerland; 2 Institute of Surgical Pathology, University Hospital Zurich, Schmelzbergstrasse 12, CH-8091 Zurich, Switzerland; 3 Institute of Pathology, Triemli Hospital, Zurich, Switzerland; 4 Institute of Pathology, Cantonal Hospital, St. Gallen, Switzerland

**Keywords:** Lung neuroendocrine tumor, Lung NET, PTEN, SUMO2/3, Small ubiquitin-related modifier protein 2/3, USP7, Herpes virus-associated ubiquitin-specific protease 7, Fluorescence in-situ hybridization, FISH, Immunohistochemistry, IHC, Tissue microarray, TMA, Small cell lung carcinoma, SCLC, Large cell neuroendocrine carcinoma, LCNEC, Typical carcinoid, TC, Atypical carcinoid, AC, Sumoylation, Ubiquitinylation, Nucleus, Cytosol, Nucleo-cytosolic partitioning

## Abstract

**Background:**

The tumor suppressor phosphatase and tensin homolog (PTEN) is a pleiotropic enzyme, inhibiting phosphatidyl-inositol-3 kinase (PI3K) signaling in the cytosol and stabilizing the genome in the nucleus. Nucleo-cytosolic partitioning is dependent on the post-translational modifications ubiquitinylation and sumoylation. This cellular compartmentalization of PTEN was investigated in lung neuroendocrine tumors (lung NET).

**Methods:**

Tumor tissues from 192 lung NET patients (surgical specimens = 183, autopsies = 9) were investigated on tissue microarrays. PTEN was H-scored by two investigators in nucleus and cytosol using the monoclonal antibody 6H2.1. Results were correlated with immunoreactivity for USP7 (herpes virus-associated ubiquitin-specific protease 7) and SUMO2/3 (small ubiquitin-related modifier protein 2/3) as well as *PTEN* and *p53* FISH gene status. Clinico-pathologic data including overall survival, proliferation rate and diagnostic markers (synaptophysin, chromogranin A, Mib-1, TTF-1) were recorded.

**Results:**

The multicentre cohort included 58 typical carcinoids (TC), 42 atypical carcinoids (AC), 32 large cell neuroendocrine carcinomas (LCNEC) and 60 small cell lung carcinomas (SCLC). Carcinoids were smaller in size and had higher synaptophysin and chromogranin A, but lower TTF-1 expressions. Patients with carcinoids were predominantly female and 10 years younger than patients with LCNEC/SCLC. In comparison to the carcinoids, LCNEC/SCLC tumors presented a stronger loss of nuclear and cytosolic PTEN associated with a loss of *PTEN* and *p53*. Concomitantly, a loss of nuclear USP7 but increase of nuclear and cytosolic SUMO2/3 was found. Loss of nuclear and cytosolic PTEN, loss of nuclear USP7 and increase of cytosolic SUMO2/3 thus correlated with poor survival. Among carcinoids, loss of cytosolic PTEN was predominantly found in TTF1-negative larger tumors of male patients. Among SCLC, loss of both cytosolic and nuclear PTEN but not proliferation rate or tumor size delineated a subgroup with poorer survival (all p-values <0.05).

**Conclusions:**

Cellular ubiquitinylation and sumoylation likely influence the functional PTEN loss in high grade lung NET. Both nuclear and cytosolic PTEN immunoreactivity should be considered for correlation with clinico-pathologic parameters.

## Background

Lung neuroendocrine tumors (NET) comprise the four histotypes typical carcinoid (TC), atypical carcinoid (AC), large cell neuroendocrine carcinoma (LCNEC) and small cell lung carcinoma (SCLC). In comparison to the carcinoids, LCNEC and SCLC are aggressive malignancies with much higher loss of growth control, due to e.g. loss of tumor suppressors, including protein phosphatase and tensin homolog (PTEN) [1,2].

The *PTEN* gene is located on chromosome 10q23.3, encoding a 403 amino acid residue protein [3]. There is no alternative protein and cells thus are ultrasensitive to subtle dosage alterations, referred to as quasi- or haploinsufficiency [4]. PTEN is a protean protein with a dual-specificity cytosolic lipid and tyrosine phosphatase activity. Both own phosphorylation status and direct protein-protein interactions are increasingly investigated [5]. Recently, a secreted PTEN Long variant was detected [6]. These pleiotropic effects are regulated by multiple layers of non-genetic regulation, including epigenetic silencing and post-transcriptional regulation by post-translational modifications (PTM) and non-coding RNAs [7].

Nuclear PTEN was originally detected by immunohistochemistry (IHC) using monoclonal antibody 6H2.1 [8]: E.g. normal pancreatic islet cells exhibited predominantly nuclear immunoreactivity, whereas endocrine pancreatic tumors had a cytosolic expression pattern [9]. This led to the concept that in normal cells PTEN is rather nuclear, but in neoplastic it is cytosolic. Various functions were attributed to nuclear PTEN, coining the term “guardian of the genome” for it. They include protein association to the centromere-specific binding protein C (CENP-C) favoring chromosomal stability, to Rad51/52 favoring DNA double strand break repair, to p300 favoring high acetylation of p53, to p73 favoring apoptosis and to the anaphase-promoting complex/cyclosome (APC/C) favoring cell cycle arrest [10-15].

The protein shuttling between nucleus and cytosol is dependent on two PTM: Ubiquitinylation and sumoylation. First, PTEN is ubiquitinylated by NEDD4-1 (neural precursor cell expressed developmentally downregulated 4–1) as the main E3 ubiquitin ligase. NEDD4-1 is regulated by cofactors NDFIP1 (NEDD4 family-interacting protein 1) and p34 [16-19]. PTEN mono-ubiquitinylation resulted in nuclear import, whereas poly-ubiquitinylation caused proteasome-mediated degradation [20]. USP7 (herpes virus-associated ubiquitin-specific protease, HAUSP) and USP13 are PTEN deubiquitinylases (DUBs) [21-23]. Second, PTEN sumoylated by small ubiquitin-related modifier proteins (SUMO) is again nuclear. Lysine residues 254 and 266 as well as the mono-ubiquitinylation site 289 in the C2 domain are SUMO acceptors [24-26] and PIASxα is a new SUMO E3 ligase [27]. No data exists so far about PTEN desumoylases but members of the SENP family are most likely involved [28].

In this study we investigated the compartmentalization of the PTEN protein in nucleus versus cytosol of lung NET in a multicenter TMA cohort together with the USP7 and the SUMO2/3 protein immunoreactivity as read-outs for cellular ubiquitinylation and sumoylation, respectively. Results were correlated with the *PTEN* and *p53* genomic status determined by fluorescence in-situ hybridization (FISH), with clinico-pathologic data including overall survival and with lung NET diagnostic markers.

## Methods

### Patients and tissue samples

One hundred and ninety-two patients with surgically resected (n = 183) or autopsy diagnosed (n = 9) neuroendocrine tumours of the lung between 1993 and 2007 at the University Hospital Zurich (n = 90), the Technical University of Munich (n = 73) and the Triemli Hospital Zurich (n = 29) were retrospectively retrieved from the computer databases and enrolled in this study. The study was approved by the Institutional Ethical Review Board of the University Hospital Zurich (reference number StV 29-2009/14).

### Tissue microarray construction

The TMA construction was accomplished with a semiautomatic tissue arrayer (Beecher Instruments, Sun Prairie, WI, USA). One or two most representative tumor areas were chosen and two tissue cores of 0.6 mm diameter assembled into the recipient paraffin blocks. Additional cores of control tissue, including normal lung as well as neuroendocrine tumors of the uterus, the ileo-caecum and the appendix were added. Four micrometer thick sections were transferred to an adhesive-coated slide system (Instrumedics, Hackensack, NJ, USA).

### Immunohistochemistry

For PTEN, the automated Leica Bond® IHC platform (Vision Biosystems, Melbourne, AUS) was used. After boiling in Tris pH 8 containing buffer H2 for 30 min, the slide was incubated for 30 min at RT with the mouse monoclonal anti-PTEN ab clone 6H2.1 (1:200 dilution, DAKO-Cytomation, Glostrup, DK). Detection was performed using the Refine-DAB Bond kit. For SUMO2/3 and USP7, the Ventana Benchmark® platform (Ventana Medical Systems, Tucson, AZ, USA) was used. The cell conditioner 1 standard mono protocol (CC1-mono) was performed: pre-treatment with boiling for 60 min in pH 8 Tris buffer following incubation with rabbit polyclonal anti-SUMO2/3 ab clone 3742 (1:500 dilution, Abcam, Cambridge, UK) or rabbit polyclonal anti-USP7 ab clone TFE1 (1:400 dilution, Bethyl Laboratories, Inc., Montgomery, TX, USA) for 60 min at RT. Detection was done with the UltraMap rabbit DAB kit. For MIB-1, synaptophysin, chromogranin A and TTF-1 our diagnostic protocols were used.

Nuclear and cytosolic immunoreactivities of PTEN, SUMO2/3 and USP7 were scored for intensity and frequency. PTEN was independently scored by two investigators (S.C. and A.S.) in a blinded manner. The intensity was semi-quantitatively scored 0 (negative), 1 (weak), 2 (moderate) or 3 (strong). The percentage of positive cells was proportionally scored 0 (0%), 10 (1-10%), 50 (11-50%) or 100 (>50%). The H-score was obtained by multiplication of intensity with percentage (range 0 to 300), summed up for the two cores and divided by two.

### Fluorescence in-situ hybridization

For *PTEN*, a dual colour probe for cytoband 10q23 and region 10p11.1-q11.1 (Vysis LSI PTEN Spectrum-Orange and CEP10 Spectrum-Green, Abbott AG, Baar, CH) was used. For *p53*, a dual colour probe for cytoband 17p13.1 (172 kb) and region 17p11.1-q11.1 (also Vysis) was used. For each case, 100 non-overlapping nuclei were evaluated using an Olympus fluorescence microscope with a 100-fold magnification objective. Tumors with <100 assessable nuclei were excluded. Normal PTEN/CEP10 and p53/CEP17 ratios were set at 1.

### Statistical analysis

Analyses were computed using the IBM SPSS 22 statistics software. Correlations of H- or FISH scores with histology or among each other were assessed by Kendall’s tau-b tests, using non-dichotomized data. Inter-observer agreement between S.C. and A.S. was controlled with Cohen’s kappa coefficient. Dunnett T3 post-hoc tests were used to assess differences in ∆H-score means between histotypes two by two. Survival data was obtained from 156 surgical patients. Patients having an OS <1 month or autopsy cases (n = 9) were excluded. Tumor-specific survival of carcinoids was not fully assessable. Markers were dichotomized closest to the median (for all tumors and for SCLC only) and OS analyzed by univariate Cox regressions and by the Kaplan-Meier method using log rank tests. A p-value <0.05 was considered significant.

## Results

### Cohort description

Hundred patients (52%) were men, 92 (48%) women. The mean age was 57 years (range 15 to 85 years). The general clinico-pathologic characteristics are summarized in Table 1. Patients with carcinoids were 10 years younger and preferentially female. In the smaller carcinoids, expression of the neuroendocrine markers synaptophysin and chromogranin A was higher, but of TTF1 lower in comparison to LCNEC/SCLC. Carcinoids (TC vs. AC) were separable by the proliferation index Mib-1 and the tumor size.

**Table 1:** **Summary of clinical data including age, sex and tumor size (Tu size) as well as relevant diagnostic immunohistochemistry markers for lung NET, including proliferation index (Mib-1), neuroendocrine (Synapto, Chrom A) and lung differentiation (TTF1)**

		Age	Sex	Tu size	Mib 1	Synapto	ChromA	TTF1
	n	mean	m/f	mean(cm)	mean(%)	any pos.	any pos.	any pos.
TC	58	53	23/35	2.2	1.6	98%	98%	45%
AC	42	49	14/28	2.8	3.1	100%	98%	48%
LCNEC	32	64	22/10	3.5	57	91%	63%	94%
SCLC	60	62	41/19	3.3	51	93%	50%	80%
All 4 histotypes	p	<0.001	<0.001	<0.001	<0.001	<0.001	<0.001	<0.001
	tau	0.208	−0.245	0.222	0.564	−0.416	−0.634	0.379
Low vs. high grade	p	<0.001	<0.001	<0.001	<0.001	<0.001	<0.001	<0.001
	tau	0.291	−0.315	0.215	0.647	−0.527	−0.723	0.467
LCNEC vs. SCLC	p	n.s.	n.s.	n.s.	n.s.	n.s.	n.s.	n.s.
	tau							

Correlation with categorized 4 histotypes, only low vs. high grade, only TC vs. AC and only LCNEC vs. SCLC. p = p-value, tau = correlation coefficient, n.s. = not significant.

### PTEN immunoreactivity in nucleus versus cytosol and FISH

**Figure 1:**
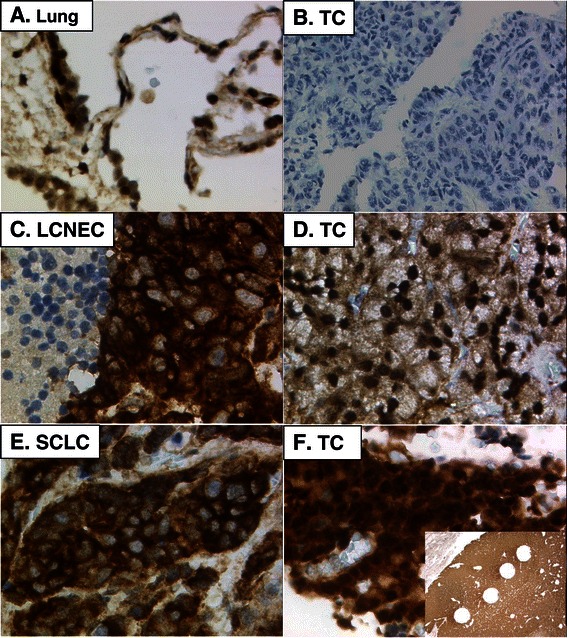
**Representative H-scores in nucleus (nucl) and cytosol (cyto) for PTEN immunoreactivity using Mab clone 6H2.1.** Original magnifications are 200-400x, except insert F (12.5x). **A.** Normal lung alveolar pneumocytes type. **B.** TC with PTEN score nucl 0 and cyto 0. **C.** LCNEC with nucl 0 and cyto 3x100. Necrotic cells on the left side were not counted. **D.** TC with nucl 3x100 and cyto 1x100. **E.** SCLC with nucl 0 and cyto 3x100. **F.** TC with nucl 3x100 and cyto 3x100. Insert: Whole section of lung TC showing homogenous PTEN staining across the tumor surface. Holes represent areas of removed tissue punches.

**Figure 2:**
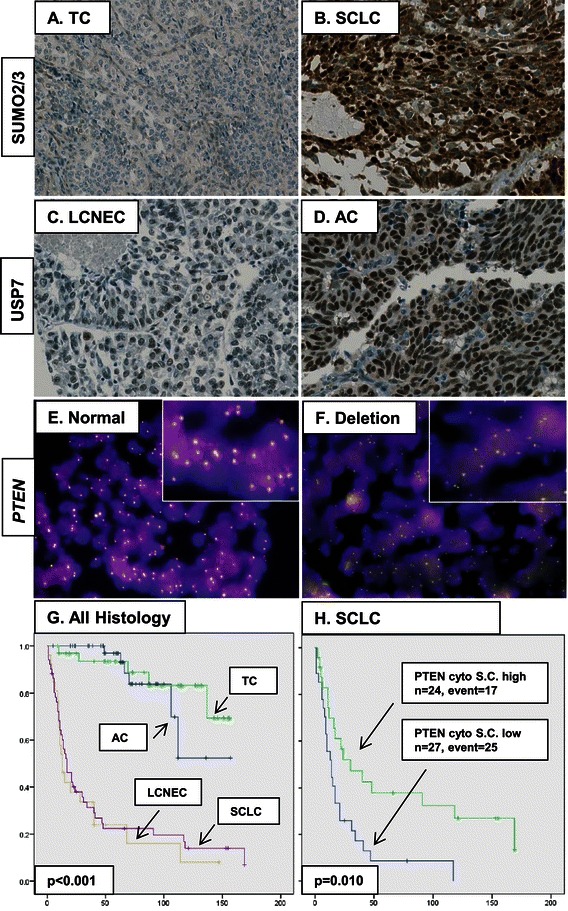
**Representative H-scores for SUMO2/3 and USP7,*PTEN*FISH examples and Kaplan-Meier curves. A.** TC with SUMO2/3 nucl 0 and cyto 0. **B.** SCLC with SUMO2/3 nucl 3x50 and cyto 2x100. **C.** LCNEC with USP7 nucl 1x50. **D.** AC with USP7 nucl 3x100. **E.** TC with normal *PTEN* status. **F.** SCLC with *PTEN* deletion. Original magnifications 200x for IHC and 630x for FISH. **G.** OS for all lung NET. **H.** OS for dichotomized cytosolic PTEN S.C. among SCLC.

The monoclonal anti-PTEN antibody clone 6H2.1 was tested on an endometrial carcinoma TMA as well as on a multi-tumor tissue and cell line microarray and was found to produce distinct nuclear and cytosolic immunoreactivity. All types of lung NET exhibited nuclear and cytosolic staining. The same was observed for SUMO2/3 but for USP7 only the nuclear signal could be scored. A minor frequency of *PTEN* deletion was found with a mean of 0.93 for the PTEN/CEP10 ratio (range 14–118). The *p53* deletion was more pronounced with a mean of 0.84 for the p53/CEP17 ratio (range 50–123). Representative IHC and FISH examples are presented in Figures 1 and 2.

### Correlation of PTEN immunoreactivity with lung NET histology

The H-score means for nuclear and cytosolic PTEN protein, correlated with histology are shown in Table 2. Robust expression was observed with a maximal H-score of 260 (range 0 to 300) in AC. Good inter-observer agreement was found with kappa values for nuclear and cytosolic H-scores of 0.710 and 0.791 for core 1, 0.684 and 0.731 for core 2, respectively. Cytosolic was always higher than nuclear PTEN expression in all histologic types. A pronounced protein loss in both compartments was observed in high grade lung NET. Differentiation among carcinoids was not possible. However, for observer S.C. a discrepancy between LCNEC and SCLC was found: SCLC showed a predominant loss of nuclear PTEN whereas in LCNEC it was predominantly cytosolic. A Dunnett T3 post hoc test showed a difference of ∆H score means between SCLC and any other histology (p < 0.001).

**Table 2:** **Summary of mean values for PTEN S.C./A.S., USP7 and SUMO2/3 protein H-score as well as PTEN and p35 FISH ratios (R) among the different neuroendocrine histotypes**

		PTEN S.C.		PTEN A.S.		USP7	SUMO2/3		*PTEN*	*p53*
		Nucl	Cyto	Nucl	Cyto	Nucl	Nucl	Cyto	FISH	FISH
	n	mean H	mean H	mean H	mean H	mean H	mean H	mean H	mean R	mean R
TC	58	218	234	168	248	226	84	72	0.96	0.90
AC	42	215	237	171	260	234	91	64	0.96	0.84
LCNEC	32	86	114	56	104	178	171	139	0.90	0.81
SCLC	60	49	163	42	146	145	152	137	0.90	0.79
All 4 histotypes	p	<0.001	<0.001	<0.001	<0.001	<0.001	<0.001	<0.001	<0.001	<0.001
	tau	−0.556	−0.314	−0.464	−0.392	−0.324	0.297	0.272	−0.149	−0.333
Low vs. high grade	p	<0.001	<0.001	<0.001	<0.001	<0.001	<0.001	<0.001	<0.001	<0.001
	tau	−0.621	−0.429	−0.540	−0.520	−0.371	0.370	0.350	−0.206	−0.307
TC vs. AC	p	n.s.	n.s.	n.s.	n.s.	n.s.	n.s.	n.s.	n.s.	<0.001
	tau									−0.280
LCNEC vs. SCLC	p	*0.006*	*0.003*	n.s.	n.s.	n.s.	n.s.	n.s.	n.s.	n.s.
	tau	−0.244	0.243							

Correlation with categorized 4 histotypes, only low vs. high grade, only TC vs. AC and only LCNEC vs. SCLC. p = p-value, tau = correlation coefficient, n.s. = not significant.

### Correlation of other markers with histology

In the high grade tumors, a decrease of nuclear USP7 as well as of the PTEN/CEP10 and p53/CEP17 ratios was found. In contrast, the expression of SUMO2/3 increased in nucleus and cytosol. Apart from *p53* deletion in TC vs. AC and nuclear or cytosolic PTEN S.C., a distinction among the low or the high grade tumors was not possible (Table 2).

### Correlation of PTEN immunoreactivity with other markers and with clinico-pathologic data for carcinoids

Irrespective of histology, loss of nuclear and cytosolic PTEN correlated with concomitant loss of USP7, *PTEN* and *p53*. Correlations of PTEN protein with SUMO2/3 were not significant or inverse (for nuclear PTEN and cytosolic SUMO2/3). However, among SCLC nuclear and cytosolic PTEN was positively correlated with cytosolic SUMO2/3 (all p-values <0.05, correlation coefficients not shown). PTEN protein was further computed against the relevant clinico-pathologic data of carcinoids in order to test for a potential subgroup (Table 3). For both investigators, a cytosolic but not nuclear loss of PTEN correlated with TTF-1 negative larger tumors of male patients.

**Table 3:** **Correlation of PTEN protein expression in nucleus and cytosol with the clinico-pathologic data for carcinoids**

		PTEN S.C.		PTEN A.S.	
Carcinoids		Nucl	Cyto	Nucl	Cyto
Age	p	n.s.	n.s.	n.s.	n.s.
	tau				
Sex(m/f)	p	n.s.	*0.012*	n.s.	*0.002*
	tau		0.222		0.291
Tumor size	p	*0.006*	*0.001*	n.s.	*<0.001*
	tau	−0.197	−0.236		−0.322
Mib-1	p	n.s.	n.s.	n.s.	n.s.
	tau				
Synaptophysin	p	*0.003*	*0.003*	n.s.	n.s.
	tau	0.301	0.317		
Chromogranin A	p	n.s.	n.s.	n.s.	n.s.
	tau				
TTF-1	p	n.s.	*0.003*	n.s.	*0.008*
	tau		0.255		0.222

p = p-value, tau = correlation coefficient, n.s. = not significant.

### Correlation of markers with overall survival

The median OS for the total cohort was 106 months (range 1 to 169 months, SE = 17.8 months). Five-year OS was 59%, for TC 97%, for AC 93.5%, for LCNEC 24% and for SCLC 22.4%. Table 4 shows the univariate Cox regression survival analyses. All markers were significant apart from TTF-1 and nuclear SUMO2/3, whereby histology showed the highest hazard ratio. As seen in Figure 2, survival for TC and AC was similar 5 to 6 years after surgery. Thereafter, AC showed a worse survival compared to TC. It is worth mentioning that the represented curves are from overall and not tumor-specific survivals. Indeed, data for tumor-specific survival was not fully assessable. The number of LCNEC with available survival (n = 21) was considered too low to compute any additional statistics. We performed a subgroup analysis among SCLC (n = 51). High expression of synaptophysin, TTF-1, cytosolic PTEN and nuclear as well cytosolic SUMO2/3 were all protective in terms of survival. Thus, loss of these markers but not increased tumor size or higher proliferation rate Mib-1 defined a subgroup of SCLC with particularly poor prognosis. In the multivariate analysis, dichotomized PTEN immunoreactivity was finally challenged against categorized histology, using TC as reference. High cytosolic but not nuclear PTEN for scorer S.C. remained significant (p-value = 0.001, HR 0.27, 95% CI 0.14-0.58), whereas the transition from TC to AC turned out not to be significant. These results favor a general protective effect of PTEN, independent of histology.

**Table 4:** **Summary of univariate Cox regression survival analyses for all tumors and only SCLC**

	All tumors (n = 156)		SCLC (n = 51)	
	p	HR	p	HR
Histology	*<0.001*	10.81		
Age	*0.010*	1.85	n.s.	
Sex	*0.001*	0.44	n.s.	
Tumor size	0.002	2.28	n.s.	
Mib-1	<0.001	6.39	n.s.	
Synaptophysin	*<0.001*	0.27	*0.042*	0.52
Chromogranin A	*<0.001*	0.13	n.s.	
TTF-1	n.s.		*0.006*	0.42
PTEN Nucl S.C.	*<0.001*	0.21	n.s.	
PTEN Nucl A.S.	*<0.001*	0.36	n.s.	
PTEN Cyto S.C.	*<0.001*	0.15	*0.010*	0.42
PTEN Cyto A.S.	*<0.001*	0.17	*0.025*	0.49
SUMO2/3 Nucl	n.s.		*0.004*	0.39
SUMO2/3 Cyto	*0.006*	1.97	*0.032*	0.51
USP7 Nucl	*<0.001*	0.27	n.s.	
PTEN FISH	*0.006*	0.44	n.s.	
p53 FISH	*0.024*	0.57	n.s.	

HR = hazard ratio, p = p-value, n.s. = not significant.

### PTEN function in nucleus and cytosol

**Figure 3:**
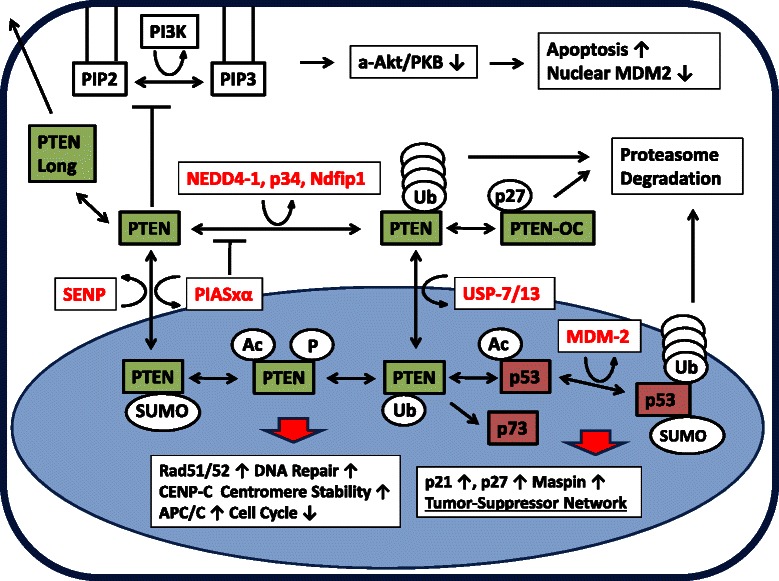
**Schematic depiction of PTEN functions in nucleus and cytosol with regard to its posttranslational modifications.** The classic pathway takes place in the cytosol underneath the plasma membrane whereby the phosphatase activity decreases the level of PIP3. The inhibition of Akt/PKB results in decreased cell proliferation and increased apoptosis. Further, a decrease of the nuclear E3 ubiquitin ligase MDM2 acting on p53 is observed. Nuclear PTEN forms a complex genome protection network by activation of Rad51/52 (DNA double strand repair), binding to CENP-C (chromosomal stability) and APC/C (slowdown of the cell cycle). It undergoes complex interactions with p53 family members. PTEN and p53 act on each other affecting acetylation and transcription. As net effect, p21, p27 and maspin are upregulated, acting as tumor suppressors in this context. PTEN sumoylation is achieved by the E3 SUMO ligase PIASxα, desumoylation most likely by a member of the SENP family. PIASxα crosstalks with the ubiquitinylation pathway. NEDD4-1 is the main E3 ubiquitin ligase regulated by cofactors p34 and NDFIP1. De-ubiquitinylation is achieved by USP7 or USP13. Poly-ubiquitinylated PTEN, p53 and MDM2 proteins are targeted for proteasome mediated degradation. Both PTEN and p53 may also be acetylated and phosphorylated to a substantial degree in the nucleus. The PTEN Long variant is secreted into the extracellular space. Cytosolic p27 is oncogenic in contrast to the nuclear moiety by e.g. retention of open conformation PTEN (PTEN-OC) in the cytoplasm targeted for proteasome degradation.

A literature review was performed on all Pubmed abstracts (2011–2014) using the term “PTEN”. Relevant data on post-translational modifications and nucleo-cytosolic partitioning is summarized in Figure 3.

## Discussion

In this study, we show that PTEN protein is expressed in both nucleus and cytosol of lung NET. In comparison to carcinoids, LCNEC/SCLC presented a protein loss in both compartments concomitant with loss of the *PTEN* and *p53* genes. The PTEN loss correlated with a loss of nuclear USP7. In contrast, high grade lung NET presented an increase of sumoylation.

There is a lack of standardization for a best practice PTEN IHC protocol and nuclear immunoreactivity as reported in endocrine pancreatic tumors and thyroid [29,30] was originally considered an artefact. In 2005, Pallares et al. tested 4 different clones on endometrial carcinomas, including a polyclonal and the monoclonals 28H6, 10P03 and 6H2.1. 6H2.1 was the only one to show a correlation between immunoreactivity and *PTEN* gene alterations such as mutation, deletion or promoter methylation [8]. This is corroborated by 2 new studies in prostate and renal cell as well as endometrial carcinoma [31,32] which propose 6H2.1 as the antibody of choice, demonstrating excellent sensitivity for both nuclear and cytoplasmic staining, specificity for PTEN immunoblot and good correlation with PTEN FISH status with regard to nuclear staining. Moreover, a recent follow-up to the Pallares study by Maiques et al. analysed the relevant analytical and preanalytical variables for PTEN IHC using 6H2.1 and DAKO-based reagents [33].

PTEN expression in normal cells such as alveolar wall pneumocytes or stromal fibroblasts is predominantly nuclear (Figure 1A), corroborating the concept that in non-neoplastic cells, the protein fulfils rather nuclear functions. Taking together the scores of 2 observers, the H-score presented a five-fold range (maximum 248 in TC, PTEN cytosol A.S. and minimum 49 in SCLC, PTEN nuclear S.C.). For a haplo-insufficient protein, this may fit well with the different behaviour of a TC versus a SCLC.

The PTEN protein loss correlated with a nuclear USP7 loss, indicating a reduction of de-ubiquitinylation, thus an increase of poly-ubiquitinylated enzyme targeted for proteasome degradation. USP7 also removes ubiquitin from p53 and the p53 E3 ubiquitin ligase MDM2 [34], therefore is a functional dose regulator of two important tumor suppressors. The regulation of USP7 in tumor cell proliferation seems to be organ-specific. In prostate carcinoma, both USP7 and MDM4 overexpression were associated with tumor aggressiveness, while both up- and down-regulation was found to inhibit colon carcinoma cell proliferation due to enhanced degradation of MDM2 following constitutively elevated p53 levels [21,35,36].

In contrast to PTEN and USP7, the expression of SUMO2/3 globally increased in the high grade tumors. These results may be explained by a concept of differential sumoylation among lung NET and/or a potential sequestration mechanism. Sequestration of nuclear PTEN was described for protein phosphatase-1 nuclear targeting subunit (PNUTS, PPP1R10) [37]. Another model indicated conformationally-dependent cytoplasmic retention and negative regulation of nuclear PTEN activity by oncogenic cytoplasmic p27Kip1 [38,39].

Both PTEN and p53 are sumoylated proteins that can be identified in SUMO-traps using SUMO interacting motifs (SIMs) [40]. PTEN undergoes complex interactions in the nucleus with p53, stimulating p300-mediated p53 acetylation following tetramerization [10] as well as with the p53 family member p73 [13]. Inversely, p53 can up- or downregulate PTEN, e.g. via caspase-mediated degradation [41,42]. p53 itself is ubiquitinylated by MDM2 [43,44]. It remains to be seen how ubiquitinylation and/or sumoylation affect PTEN-p53 interaction. There is also crosstalk between sumoylation and ubiquitinylation: E.g. the SUMO E3 ligase PIASxα enhanced PTEN protein stability by reducing its ubiquitinylation [27] and PTEN-SUMO1 showed a reduced capacity to form covalent interactions with mono-ubiquitin [25].

How the post-translationally modified PTEN protein, including PTEN-SUMO, −Ub and potentially -Ac, −P or -OC (open conformation)-p27 shuttles between nucleus and cytosol is unclear [45,46]. The protein lacks a true nuclear localization signal. Different mechanisms were proposed for this shuttling including simple diffusion through nuclear pores [47], active RAN-mediated nuclear import [48] and transport via the major vault protein (MVP) [49].

The histopathologic diagnosis between TC and AC is notoriously difficult to be made. Indeed, there is a trend to pool them into carcinoids and secondarily stratify them according to molecular data. This view is corroborated by the similar survivals curves on Figure 2. In our opinion, the entity “atypical carcinoid” may simply arise by the fact that enlarging carcinoids have a higher mitotic rate and more necrotic foci. These data need however to be interpreted with caution, since OS and not tumor-specific survival was computed. This is of significant importance for mainly indolent tumors such as carcinoids. For this same reason, we did not perform a subgroup analysis among carcinoids. However, the correlation with the other clinico-pathologic parameters showed that a PTEN loss is primarily found in male with TTF-1 negative larger carcinoids.

Differences between LCNEC and SCLC are also debated. SCLC cells have a size less than the diameter of 3 small resting lymphocytes, but interspersed larger elements are often observed. All markers apart nuclear and cytosolic PTEN of scorer S.C. failed to distinguish them and no survival differences were found. The survival results favour a concept of single high-grade lung NET [50]. Among SCLC, we identified a subset in which high cytosolic PTEN, high nuclear and cytosolic SUMO2/3, high synaptophysin and high TTF1 were associated with better survival. As observed in the carcinoids as well, these results are best interpreted as tumor dedifferentiation being associated with loss of respective tumor suppressor and differentiation markers.

From a therapeutic point of view, loss of PTEN leaves cells sensitive to DNA damage, but it also provides a PI3K pathway survival signal, the inhibition of which could kill the tumor [51]. This concept has created considerable oncologic interest since numerous PI3K inhibitors are currently investigated and may be combined with DNA damaging agents. It is conceivable that PTEN PTM interfere with PI3K inhibition via determination of the cytosolic enzyme activity. A further question is to what degree such PTM would affect the ratio between intracellular PTEN and its secreted variant PTEN Long that may be bestowed to cancer cells from stroma or introduced biopharmaceutical.

## Conclusion

In summary, cellular ubiquitinylation and sumoylation likely influence the functional PTEN loss in high grade lung NET. Both nuclear and cytosolic PTEN immunoreactivity should be considered for correlation with clinico-pathologic parameters.
